# Supplementary material for: Long non-coding RNA LncKdm2b regulates cortical neuronal differentiation by cis-activating Kdm2b
Source: Protein Cell. 2019 Jul 17;11(3):161–86. doi: 10.1007/s13238-019-0650-z (PMC7026249; doi:10.1007/s13238-019-0650-z)
Supplement: Supplementary file 1 — Supplementary material 1 (PDF 5090 kb) [file 13238_2019_650_MOESM1_ESM.pdf]

Supplementary Information

Supplementary Materials and Methods

Key resources table

REAGENT or RESOURCE	SOURCE	IDENTIFIER
**Antibodies**		
Mouse monoclonal to β-Actin	Chemicon	MAB1501; RRID: AB_2223041
Mouse monoclonal to TUJ1	Sigma	T8660; RRID: AB_477590
Rat monoclonal to BrdU	Abcam	ab6326; RRID: AB_305426
Mouse monoclonal to BrdU	Santa Cruz	sc-32323, RRID: AB_626766
Rabbit monoclonal to Cleaved Caspase-3	Cell Signaling	9664; RRID: AB_2070042
Rat monoclonal to CTIP2	Abcam	ab18465; RRID: AB_2064130
Rabbit polyclonal to GFP	Invitrogen	A11122; RRID: AB_221569
Rabbit polyclonal to KDM2B	Millipore	17-10264; RRID: AB_11205420
Rabbit polyclonal to KDM2B	Millipore	09-864; RRID: AB_10806072

Rabbit polyclonal to KDM2B	Labaratory of JieKai Chen	N/A
Rabbit polyclonal to PAX6	Chemicon	AB2237; RRID: AB_1587367
Rabbit polyclonal to TBR2	Abcam	ab23345; RRID: AB_778267
Rabbit polyclonal to SOX2	Milipore	ab5603; RRID: AB_2286686
Rabbit polyclonal to NeuN	Abcam	ab177487
Rabbit polyclonal to GFAP	DAKO	Z0334
Mouse monoclonal to FLAG	Sigma-Aldrich	F1804; RRID: AB_262044
Mouse monoclonal to SATB2	Abcam	ab51502; RRID: AB_882455
Goat polyclonal to UNC5D	R&D	AF1429; RRID: AB_2304199
Sheep anti-DIG AP	Roche	11093274910; RRID: AB_514497
Mouse monoclonal to H3K4me3	Active Motif	MABI0304; RRID: AB_514497
Mouse monoclonal to H3K27ac	Millipore	17-683; RRID: AB_1977529

Rabbit polyclonal to NEUROD2	Abcam	ab104430; RRID: AB_10975628
Rabbit polyclonal to SATB1	Abclonal	A5800
Mouse monoclonal to hnRNPAB	Santa Cruz	sc-32323
Rabbit polyclonal to GAPDH	Cwbio	CW0101M; RRID: AB_2665434
Rabbit polyclonal to β-TUBULIN	Proteintech	10094-1-AP; RRID: AB_2210695
**Chemicals, Peptides, and Recombinant Proteins**		
B27	Thermo Fisher	17504044
N2	Thermo Fisher	17502048
hEGF	Thermo Fisher	PHG0311
hFGF2	Thermo Fisher	PHG0261
Papain	Wortington	LS003118
DNase I	Sigma-Aldrich	DN-25
DMEM-F12 medium	Thermo Fisher	12634-010
Protein G agarose		
Streptavidin Agarose	Thermo Fisher	S951
5-Bromouridine 5′-triphosphate	Sigma-Aldrich	B7166
Mung Bean Nuclease	Takara Bio	2420A
Micrococcal Nuclease	NEB	M0247S

Vanadyl Ribonucleoside Complex	Sangon Biotech	B644221
Protease inhibitor	Biotool	B14001
PMSF	Sigma-Aldrich	P7626
Proteinase K	Sigma	P4032
NBT/BCIP	Roche	11681451001
DIG-NTP	Roche	11277073910
Biotin RNA labeling mix	Roche	11685597912
CDP-star	Roche	11685627001
paraformaldehyde	Sigma-Aldrich	P6148
FastGreen	Sigma-Aldrich	F7252
Lipofectamine 3000	Invitrogen	L3000-150
Trizol	Thermo Fisher	15596026
**Bacterial and Virus Strains**		
E. coli DH5α	TransGen	CD201-01
E. coli Stbl3	TransGen	CD521-01
Mus musculus BAC clone	BACPAC	RP23-214I6
Lentivirus vector, pLKO.1-zsGreen		
**Critical Commercial Assays**		
Mouse Neural Stem Cell Nucleofector® Kit	LONZA	VAPG-1004

HiScribe™ T7 High Yield RNA Synthesis Kit	NEB	E2040S
**Experimental Models: Cell Lines**		
Mouse Neuro-2a cell line	The Cell Bank of Chinese Academy of Sciences	TCM29
Mouse NE-4C cell line	The Cell Bank of Chinese Academy of Sciences	SCSP-1501
Human HEK293T cell line	A gift from Dr. Hongbing Shu	
LncKdm2b polyA Knock-in mouse ES cells	This paper	N/A
LncKdm2b polyA Knock-in and *Kdm2b* indels Mouse ES Cells	This paper	N/A
**Experimental Models: Organisms/Strains**		
Mouse: CD-1	Hunan Laboratory Animal Co	SJA

Mouse: C57BL/6	Hunan Laboratory Animal Co	SJA
Mouse: *Ai14* reporter	(Madisen et al., 2010)	
Mouse: *Kdm2b* $^{CreERT2/+}$	This paper	N/A
**Recombinant DNA**		
pGEM-Teasy	Promega	A1360
pCAGGS		
pCAG-mir30		
pMD2.G	Addgene	12259
psPAX2	Addgene	12260
pGL3-basic	Promega	E1751
phRL-TK	Promega	E6921
pCALNL-DsRed	Addgene	13769
**Software and Algorithms**		
DAVID functional annotation tool	(Huang da et al., 2009)	https://david.ncifcrf.gov/
UCSC Genome Browser	(Kent et al., 2002)	http://genome.ucsc.edu/

CPAT	(Wang et al., 2013)	http://lilab.research.bcm.edu/cpat/index.php/
PhyloCSF	(Lin et al., 2011)	http://compbio.mit.edu/PhyloCSF/
RNAfold web server		http://rna.tbi.univie.ac.at/
Prism	GraphPad	Ver 6

Supplementary tables

Table S1. Divergent lncRNAs identified in this study.

Table S2. Significantly-enriched proteins in *LncKdm2b*-precipitating extracts compared to the antisense-*LncKdm2b*.

Table S3. Statistical analyses of electroporated cortices.

Table S4. Sequences for all primers used in this study.

Supplementary figure titles and legends

Figure S1. *LncKdm2b* and *Kdm2b* are transiently expressed in the developing mouse embryonic cortex.

(A) Gene ontology (GO) analysis of coding genes associated with divergent lncRNAs. The top GO terms (>1.5-fold and P < 1×10^{-6}) are shown.

(B) Protein-coding scores of *LncKdm2b* using CPAT and PhyloCSF programs.

(C) Thirteen putative ORFs of *LncKdm2b* were cloned into the pFLAG-N3 vector with

3 × Flag tag sequence fused to their 3’ for HEK293T cell transfection. After 48 hours, immunoblotting was performed to detect Flag-tagged proteins. PRRX1B tagged with 3 × Flag tag served as a positive control. Data are representative of three independent experiments.

(D) Fragments per kilobase per million mapped reads (FPKM) values for *LncKdm2b*, and *Kdm2b* in mouse ESCs, mouse NPCs and mouse cortices at indicated developmental stages.

(E-L) RT-qPCR analyses of indicated markers of E10.5, E12.5, E14.5 and adult (6 weeks) mouse dorsal forebrains. The y-axis represents relative expression normalized to *Gapdh*.

(M) Representative immunoblotting of mouse dorsal forebrains using antibodies against KDM2B and β-TUBULIN.

(N) Northern blots of *LncKdm2b* and *Gapdh* using poly(A) RNAs extracted from mouse dorsal forebrains.

(O) Schematic illustration of *in situ* hybridization probes for mouse *LncKdm2b* and *Kdm2b*.

(P) *In situ* hybridization (ISH) of *LncKdm2b* on coronal sections of E12.5 mouse forebrain (the CD-1 strain). Scale bars, 100 μm. The higher-magnification image of the boxed area shows immunofluorescent staining for TBR2 (green) and TUJ1 on ISH section of *LncKdm2b* (red). Scale bars, 50 μm.

(Q) Southern blot analysis of genomic DNA from wild-type (WT) or $Kdm2b^{CreERT2/+}$

knock-in mice.

(R-S) Immunofluorescent staining for EGFP (green), TBR2 (red), TUJ1 (blue), UNC5D (red), and DAPI (blue) on cortical sections of E14.5 *Kdm2b*$^{CreERT2/+}$ mice. Boxed areas are enlarged at the bottom-right corners. Scale bars, 50 μm.

In (E-L), quantification data are shown as mean + SD (n = 3 unless otherwise indicated).

Ctx, cortex; LV, lateral ventricl; VZ, ventricular zone; SVZ, subventricular zone; IZ, intermediate zone.

Figure S2. *Kdm2b*-expressing cortical cells are fated to be cortical projection neurons.

(A-D) Immunofluorescent staining for SATB2 and CTIP2 on coronal cortical sections of E16.5 (A) and P0 (C) *Kdm2b*$^{CreERT2/+}$*;Ai14* embryos. Tamoxifen (TAM, 100 mg/kg) was injected at E12.5 or E14.5 respectively. Boxed areas are enlarged at the bottom-right corners. Quantification of SATB2 or CTIP2 expression in tdTomato^{+} recombined cells (B and D). A total of 3372 cells from 2 embryos were analyzed in (B), and 2282 cells from 2 animals in (D).

(E) Immunofluorescent staining for SOX2 and TUJ1 on head sections of E10.5 *Kdm2b*$^{CreERT2/+}$*;Ai14* embryo. Boxed areas are enlarged at the bottom-right corners.

(F) Immunofluorescent staining for PAX6 (top) and TBR2 (bottom) on head sections of E13.5 *Kdm2b*$^{CreERT2/+}$*;Ai14* embryo. Tamoxifen (TAM, 100 mg/kg) was injected at E12.5. Boxed areas are enlarged at the bottom-right corners.

(G-H) Immunofluorescent staining for SATB2 and CTIP2 on coronal cortical sections of P7 *Kdm2b*$^{CreERT2/+}$*;Ai14* mouse brain. Boxed areas are enlarged at the bottom-right corners. Quantification of SATB2 or CTIP2 expression in tdTomato^{+} recombined cells (H). A total of 373 cells were analyzed.

In (B), (D), and (H), quantification data are shown as mean + SEM.

LV, lateral ventricle; MB, midbrain. Scale bars, 50 μm.

Figure S3. *LncKdm2b* maintains *Kdm2b* transcription in *cis*.

(A) Percentages of marker-expressing neuronal cells in adherent cultures derived from E12.5 cortices.

(B) RT-qPCR analysis of *LncKdm2b* and *Kdm2b* RNA levels in adherent cultures derived from E12.5 cortices. The cultures were treated with indicated ASOs. The y-axis represents relative expression normalized to *Gapdh*.

(C) RT-qPCR analysis of *Zfp292* mRNA levels in Neuro-2a cells treated with indicated *LncKdm2b* ASOs for three days. The y-axis represents relative expression normalized to *Gapdh*.

(D) Genotyping of NE-4C clones with *LncKdm2b*'s exon2 knocked out.

(E) RT-qPCR analysis of *Zfp292* mRNA levels in NE-4C clones with *LncKdm2b*'s exon2 knocked out. The y-axis represents relative expression normalized to *Gapdh*.

(F) RT-qPCR analysis for *LncKdm2b*, *Gapdh*, *Actb*, *Xist*, and *Neat2* from cytoplasmic and nuclear RNA fractions of primary E14.5 cortical neural progenitor cells (NPCs)

cultured *in vitro* for four days.

(G) Fluorescent *in situ* hybridization of *LncKdm2b* on cortical NPCs treated with or without RNase A. The nuclei were counter-stained with DAPI.

(H-I) RT-qPCR analysis of *LncKdm2b* and *Kdm2b* mRNA levels in Neuro-2a and NE-4C cells transfected with empty or *LncKdm2b*-expressing vectors for 48 hours. The y-axis represents relative expression normalized to *Gapdh* (n = 5).

(J) RT-qPCR analysis of *LncKdm2b* and *Kdm2b* mRNA levels in parental and *LncKdm2b*-KO NE-4C cells transfected with empty or *LncKdm2b*-expressing vectors for 48 hours. The y-axis represents relative expression normalized to *Gapdh*.

(K) RT-qPCR analysis of *LncKdm2b* and *Kdm2b* RNA levels in Neuro-2a cells treated for two days with Scramble ASOs or ASOs targeting *Kdm2b*.

In (A) and (H-I), quantification data are shown as mean + SEM. In (B–C), (E-F), and (J-K) quantification data are shown as mean + SD (n = 3 unless otherwise indicated). In (B) and (H-I), statistical significance was determined using 2-tailed Student's *t* test. In (C), (E), and (J-K), statistical significance was determined using 1-way ANOVA followed by the Turkey's *post hoc* test. *$p < 0.05$, **$p < 0.01$, ***$p < 0.001$, "NS" indicates no significance.

Figure S4. *LncKdm2b* maintains *Kdm2b* transcription in *cis*.

(A) Relative crosslinking frequency between the T5 and *Kdm2b*'s TSS measured by 3C-qPCR in Neuro-2a cells treated for two days with Scramble ASOs or ASOs

targeting *LncKdm2b*. The y-axis shows fold enrichment normalized to the scramble control.

(B) Luciferase activities in experiments where indicated vectors were transfected into HEK293T cells for 24 hours. ‘Forward’ and ‘Reverse’ indicate directions same as or opposite of *Kdm2b*’s transcription orientation.

(C) Genotyping of NE-4C cells with the T5 region knocked out.

(D) RT-qPCR analysis of *Zfp292* mRNA levels in NE-4C clones with the T5 region knocked out. The y-axis represents relative expression normalized to *Gapdh*.

(E) Genotyping of cortical cells subjected to Cas9-mediated knockout of the T5 region.

In (A-B) and (D), quantification data are shown as mean + SD (n = 3 unless otherwise indicated). In (A-B) and (D), statistical significance was determined using 1-way ANOVA followed by the Turkey’s *post hoc* test. *$p < 0.05$, **$p < 0.01$, ***$p < 0.001$, “NS” indicates no significance.

Figure S5. Characterization of *LncKdm2b*-associated proteins.

(A) The illustration describing the Gal4-λN/*BoxB* RNA tethering system.

(B-D) Relative luciferase activities in experiments where Neuro-2a cells were transfected with plasmids expressing *BoxB*-tagged *LacZ*, full-length *LncKdm2b*, 5’ *LncKdm2b*, or 3’ *LncKdm2b* along with Gal4-λN and 5×UAS-TK-Luciferase-expressing plasmids for 24 hours.

(E) RT-qPCR analysis of *Kdm2b* mRNA levels in Neuro-2a cells treated for three days

with scramble siRNA (siNC) or siRNA targeting indicated molecules. The y-axis represents relative expression normalized to *Gapdh*.

(F) Fluorescent *in situ* hybridization of *LncKdm2b* on cortical NPCs followed by co-staining of hnRNPAB and DAPI.

(G) RNA secondary structure prediction by *RNAfold* showed two putative stem-loop structures.

(H) ChIP-qPCR analysis of indicated primer sets enriched by anti-hnRNPAB antibodies in Neuro-2a cells. The y-axis shows fold enrichment normalized to the IgG control.

In (B–E) and (H), quantification data are shown as mean + SD (n = 3 unless otherwise indicated). In (B–E), statistical significance was determined using 1-way ANOVA followed by the Turkey's *post hoc* test. In (H), statistical significance was determined using 2-tailed Student's t test. *$p < 0.05$, **$p < 0.01$, ***$p < 0.001$, "NS" indicates no significance.

Figure S6. *Kdm2b* promotes cortical neurogenesis.

(A) Representative immunoblots of HEK293T cells transfected with empty vector or KDM2B-expressing vector for two days using antibodies against KDM2B and ACTIN.

(B) Immunoblotting of E15.5 embryonic cortices with indicated genotypes using antibodies against KDM2B and β-TUBULIN.

(C-D) Quantification of relative location (C) and percentiles (D) of $NEUROD2^+$ transduced cells in scramble or KDM2B shRNA electroporated sections. Three embryos each.

(E-F) E13.5 mouse cortices were electroporated with indicated combination of vectors, with transduced cells labeled with EGFP. Embryos were sacrificed at E16.5 for immunofluorescent analysis. Representative VZ/SVZ images of sections immunostained with PAX6 (E) and quantification of $PAX6^+$ transduced cells (F) were shown. Three embryos in control and shKDM2B, five embryos in shKDM2B plus KDM2B. Scale bars, 50 μm.

(G) Quantification of Cleaved $Caspase3^+$ transduced cells in scramble or KDM2B shRNA electroporated sections. Three embryos each.

In (C-D) and (F-G), quantification data are shown as mean + SEM. In (C-D) and (G), statistical significance was determined using 2-tailed Student's *t* test. In (F), statistical significance was determined using1-way ANOVA followed by the Turkey's *post hoc* test. *$p < 0.05$, **$p < 0.01$, ***$p < 0.001$, "NS" indicates no significance.

VZ, ventricular zone; SVZ, subventricular zone; IZ, intermediate zone; CP, cortical plate.

Figure S7. *LncKdm2b* maintains mouse cortical neurogenesis through KDM2B.

(A) E13.5 mouse cortices were electroporated with Scramble ASO or ASOs targeting *LncKdm2b*, with transduced cells labeled with EGFP. RT-qPCR analysis of *LncKdm2b*

and *Kdm2b* mRNA levels in $EGFP^+$ and EGFP- cells from E15.5 electroporated cortices. The y-axis represents relative expression normalized to *Gapdh*.

(B) Quantification of Cleaved $Caspase3^+$ transduced cells in Scramble or *LncKdm2b* ASO electroporated cortices. Three embryos each.

(C-E) E13.5 mouse cortices were electroporated with indicated siRNAs, with transduced cells labeled with EGFP. Embryos were sacrificed at E16.5 for PAX6 immunofluorescent stainings (C). The relative location of $EGFP^+$ cells (D) and percentiles of $PAX6^+$ transduced cells (E) were quantified. Three embryos in control (siNC), five embryos in sihnRNPA2B1. Scale bars, 50 μm.

(F-G) E13.5 mouse cortices were electroporated with empty or *LncKdm2b*-expressing vector, along with mCherry-expressing vector to label transduced cells. Embryos were sacrificed at E15.5 followed by DAPI staining of coronal sections (F). The locations of $mCherry^+$ cells were quantified (G). Seven embryos each. Scale bars, 50 μm.

In (A), quantification data are shown as mean + SD. In (B), (D-E), and (G), quantification data are shown as mean + SEM. In (A), statistical significance was determined using 2-way ANOVA followed by the Bonferroni's *post hoc* test. In (B), (D-E), and (G), statistical significance was determined using 2-tailed Student's *t* test. *$p < 0.05$, **$p < 0.01$, ***$p < 0.001$, "NS" indicates no significance.

VZ, ventricular zone; SVZ, subventricular zone; IZ, intermediate zone; CP, cortical plate.

Figure S8. *LncKdm2b* regulates cortical neuronal differentiation and migration.

(A) A schematic illustration of the piggyBac-CRISPR/Cas9 toolkit for *LncKdm2b* knockout by *in utero* electroporation. Two sgRNAs were used for deletion of the *LncKdm2b*'s second exon.

(B) Quantification of neuron to glia ratio in brains at P10. Four brains each.

(C) Quantification of the distribution of $SATB2^{+}EGFP^{+}$ cells in cortices at P10. Four brains each.

(D) Quantification of Cleaved $Caspase3^{+}$ transduced cells. Three brains each.

In (B-D), quantification data are shown as mean + SEM. In (B-D), statistical significance was determined using 2-tailed Student's *t* test. *$p < 0.05$, **$p < 0.01$, ***$p < 0.001$, "NS" indicates no significance.

REFERENCES

Huang da, W., Sherman, B.T., and Lempicki, R.A. (2009) Systematic and integrative analysis of large gene lists using DAVID bioinformatics resources. Nat Protoc 4:44-57

Kent, W.J., Sugnet, C.W., Furey, T.S., Roskin, K.M., Pringle, T.H., Zahler, A.M., and Haussler, D. (2002) The human genome browser at UCSC. Genome Res 12:996-1006

Lin, M.F., Jungreis, I., and Kellis, M. (2011) PhyloCSF: a comparative genomics method to distinguish protein coding and non-coding regions. Bioinformatics 27:i275-282

Madisen, L., Zwingman, T.A., Sunkin, S.M., Oh, S.W., Zariwala, H.A., Gu, H., Ng, L.L., Palmiter, R.D., Hawrylycz, M.J., Jones, A.R.*, et al.* (2010) A robust and high-throughput Cre reporting and characterization system for the whole mouse brain. Nat Neurosci 13:133-140

Wang, L., Park, H.J., Dasari, S., Wang, S., Kocher, J.P., and Li, W. (2013) CPAT: Coding-Potential Assessment Tool using an alignment-free logistic regression model. Nucleic Acids Res 41:e74

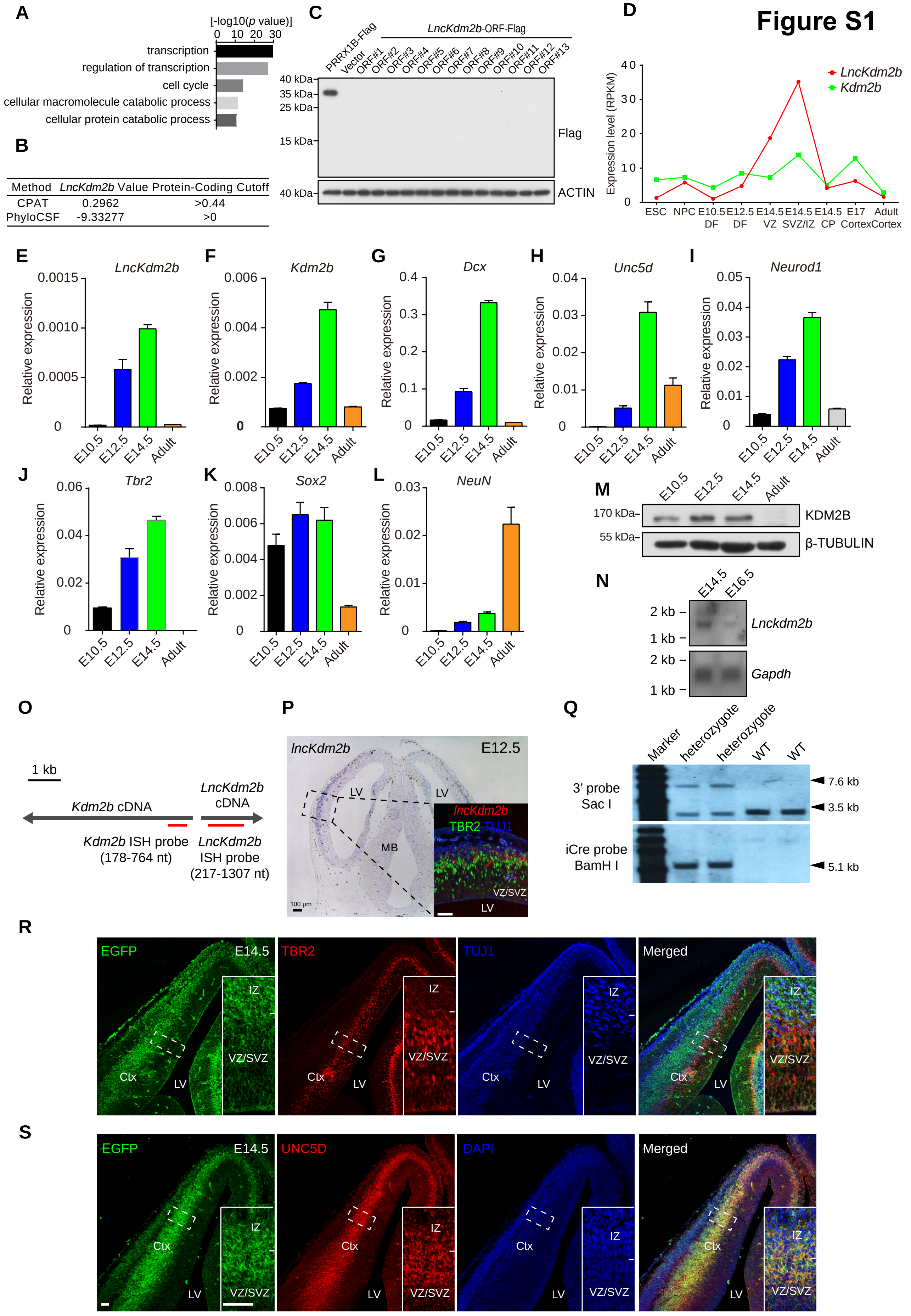

Figure S1
A
[-log10(p value)]
0 10 20 30
transcription
regulation of transcription
cell cycle
cellular macromolecule catabolic process
cellular protein catabolic process
B
Method LncKdm2b Value Protein-Coding Cutoff
CPAT 0.2962 >0.44
PhyloCSF -9.33277 >0
C
LncKdm2b-ORF-Flag
PRRX1B-Flag
Vector
ORF#1 ORF#2 ORF#3 ORF#4 ORF#5 ORF#6 ORF#7 ORF#8 ORF#9 ORF#10 ORF#11 ORF#12 ORF#13
40 kDa
35 kDa
25 kDa
15 kDa
Flag
40 kDa
ACTIN
D
LncKdm2b
Kdm2b
Expression level (RPKM)
ESC NPC E10.5 DF E12.5 DF E14.5 VZ E14.5 SVZ/IZ E14.5 CP E17 Cortex Adult Cortex
E
LncKdm2b
F
Kdm2b
G
Dcx
H
Unc5d
I
Neurod1
Relative expression
E10.5 E12.5 E14.5 Adult
J
Tbr2
K
Sox2
L
NeuN
M
E10.5 E12.5 E14.5 Adult
170 kDa
KDM2B
55 kDa
β-TUBULIN
N
E14.5 E16.5
2 kb
1 kb
Lnckdm2b
Gapdh
O
1 kb
Kdm2b cDNA
LncKdm2b cDNA
Kdm2b ISH probe (178-764 nt)
LncKdm2b ISH probe (217-1307 nt)
P
lncKdm2b
E12.5
LV
MB
100 μm
lncKdm2b
TBR2 TUJ1
VZ/SVZ
Q
Marker heterozygote heterozygote WT WT
3' probe Sac I
iCre probe BamH I
7.6 kb
3.5 kb
5.1 kb
R
EGFP E14.5
TBR2
TUJ1
Merged
IZ
VZ/SVZ
Ctx
LV
S
EGFP E14.5
UNC5D
DAPI
Merged

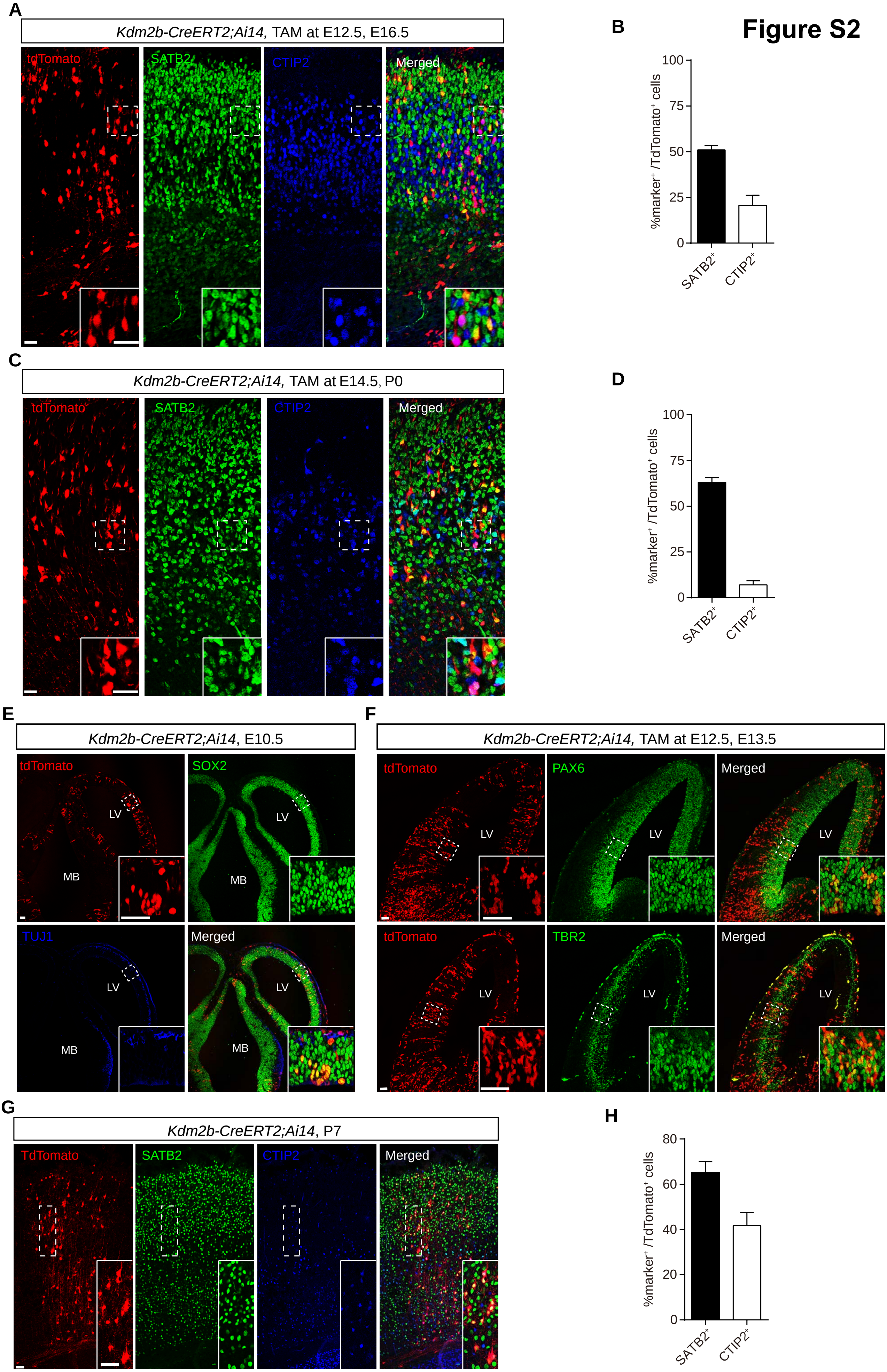
Figure S2
A
Kdm2b-CreERT2;Ai14, TAM at E12.5, E16.5
tdTomato
SATB2
CTIP2
Merged
B
%marker⁺ /TdTomato⁺ cells
100
75
50
25
0
SATB2⁺
CTIP2⁺
C
Kdm2b-CreERT2;Ai14, TAM at E14.5, P0
tdTomato
SATB2
CTIP2
Merged
D
%marker⁺ /TdTomato⁺ cells
100
75
50
25
0
SATB2⁺
CTIP2⁺
E
Kdm2b-CreERT2;Ai14, E10.5
tdTomato
SOX2
TUJ1
Merged
LV
MB
F
Kdm2b-CreERT2;Ai14, TAM at E12.5, E13.5
tdTomato
PAX6
Merged
TBR2
LV
G
Kdm2b-CreERT2;Ai14, P7
TdTomato
SATB2
CTIP2
Merged
H
%marker⁺ /TdTomato⁺ cells
80
60
40
20
0
SATB2⁺
CTIP2⁺

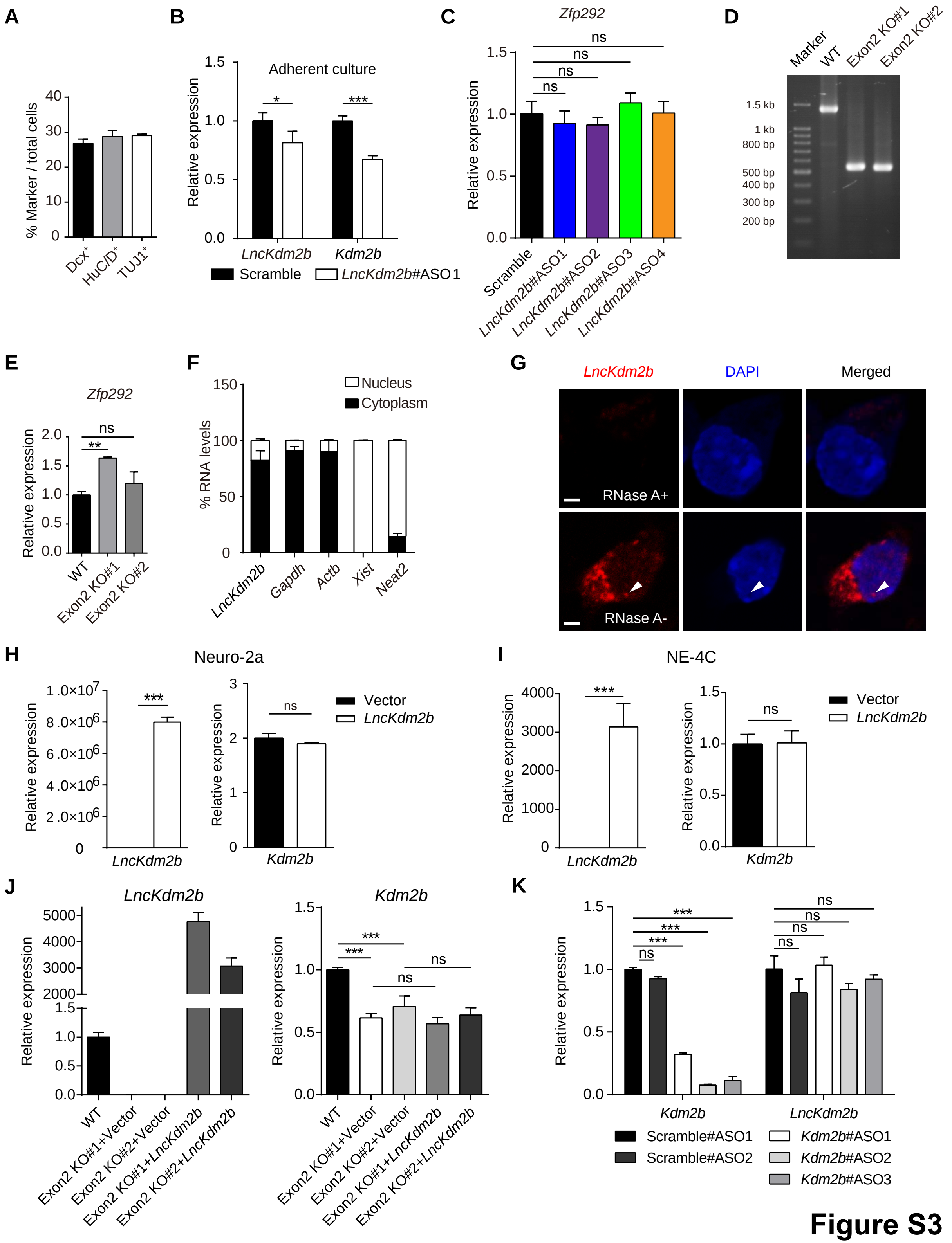

A
% Marker / total cells
Dcx+
HuC/D+
TUJ1+
B
Adherent culture
Relative expression
LncKdm2b
Kdm2b
Scramble
LncKdm2b#ASO1
C
Zfp292
ns
Scramble
LncKdm2b#ASO1
LncKdm2b#ASO2
LncKdm2b#ASO3
LncKdm2b#ASO4
D
Marker
WT
Exon2 KO#1
Exon2 KO#2
1.5 kb
1 kb
800 bp
500 bp
400 bp
300 bp
200 bp
E
Zfp292
F
Nucleus
Cytoplasm
% RNA levels
Gapdh
Actb
Xist
Neat2
G
DAPI
Merged
RNase A+
RNase A-
H
Neuro-2a
Vector
I
NE-4C
J
WT
Exon2 KO#1+Vector
Exon2 KO#2+Vector
Exon2 KO#1+LncKdm2b
Exon2 KO#2+LncKdm2b
K
Scramble#ASO1
Scramble#ASO2
Kdm2b#ASO1
Kdm2b#ASO2
Kdm2b#ASO3

Figure S3

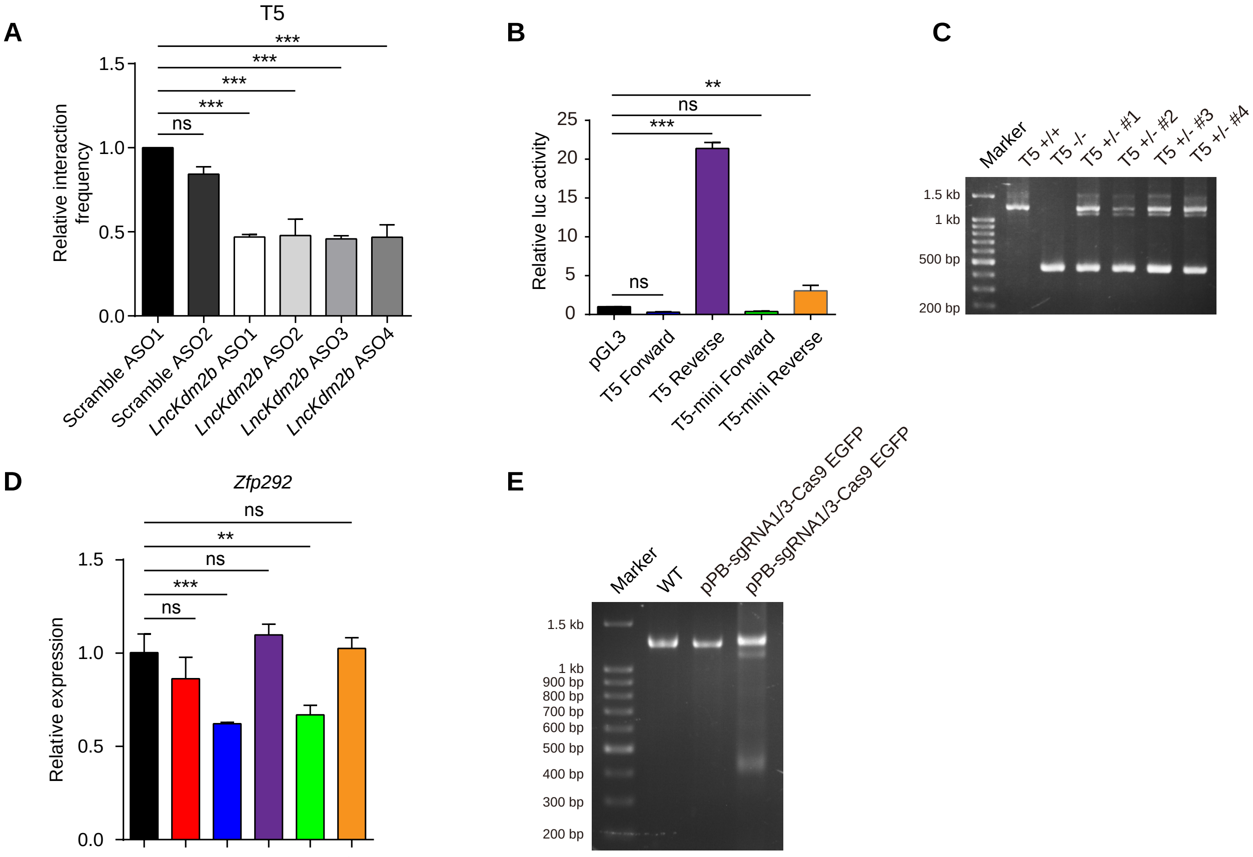
A
T5
Relative interaction frequency
Scramble ASO1
Scramble ASO2
LncKdm2b ASO1
LncKdm2b ASO2
LncKdm2b ASO3
LncKdm2b ASO4
B
Relative luc activity
pGL3
T5 Forward
T5 Reverse
T5-mini Forward
T5-mini Reverse
C
Marker
T5 +/+
T5 -/-
T5 +/- #1
T5 +/- #2
T5 +/- #3
T5 +/- #4
1.5 kb
1 kb
500 bp
200 bp
D
Zfp292
Relative expression
E
Marker
WT
pPB-sgRNA1/3-Cas9 EGFP
pPB-sgRNA1/3-Cas9 EGFP
1.5 kb
1 kb
900 bp
800 bp
700 bp
600 bp
500 bp
400 bp
300 bp
200 bp

Figure S4

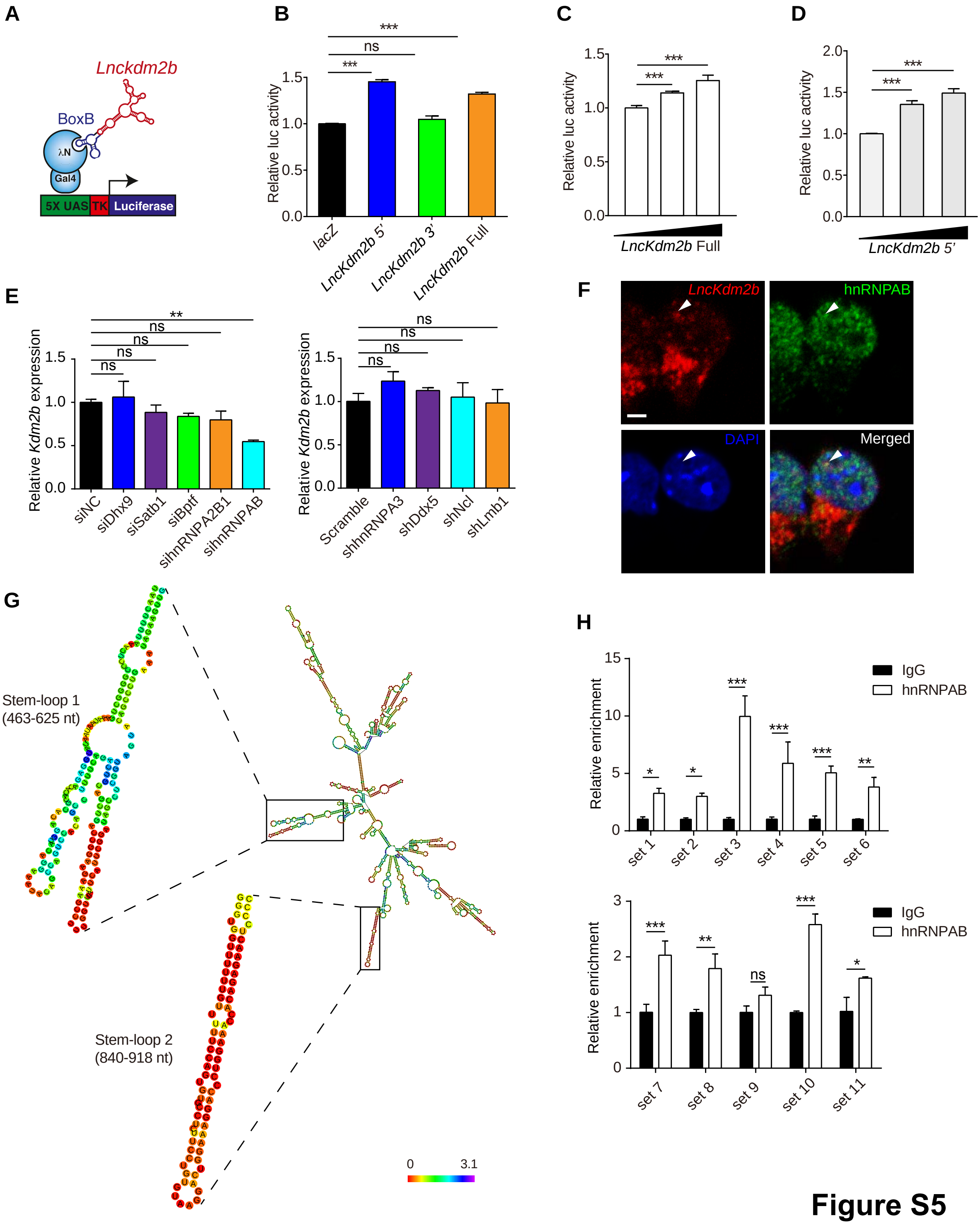
A
Lnckdm2b
BoxB
λN
Gal4
5X UAS
TK
Luciferase
B
Relative luc activity
***
ns
***
1.5
1.0
0.5
0.0
lacZ
LncKdm2b 5'
LncKdm2b 3'
LncKdm2b Full
C
Relative luc activity
***
***
1.5
1.0
0.5
0.0
LncKdm2b Full
D
Relative luc activity
***
***
2.0
1.5
1.0
0.5
0.0
LncKdm2b 5'
E
Relative Kdm2b expression
**
ns
ns
ns
ns
1.5
1.0
0.5
0.0
siNC
siDhx9
siSatb1
siBptf
sihnRNPA2B1
sihnRNPAB
Relative Kdm2b expression
ns
ns
ns
ns
1.5
1.0
0.5
0.0
Scramble
shhnRNPA3
shDdx5
shNcl
shLmb1
F
LncKdm2b
hnRNPAB
DAPI
Merged
G
Stem-loop 1
(463-625 nt)
Stem-loop 2
(840-918 nt)
0
3.1
H
Relative enrichment
15
10
5
0
IgG
hnRNPAB
*
*
***
***
***
**
set 1
set 2
set 3
set 4
set 5
set 6
Relative enrichment
3
2
1
0
IgG
hnRNPAB
***
**
ns
***
*
set 7
set 8
set 9
set 10
set 11

Figure S5

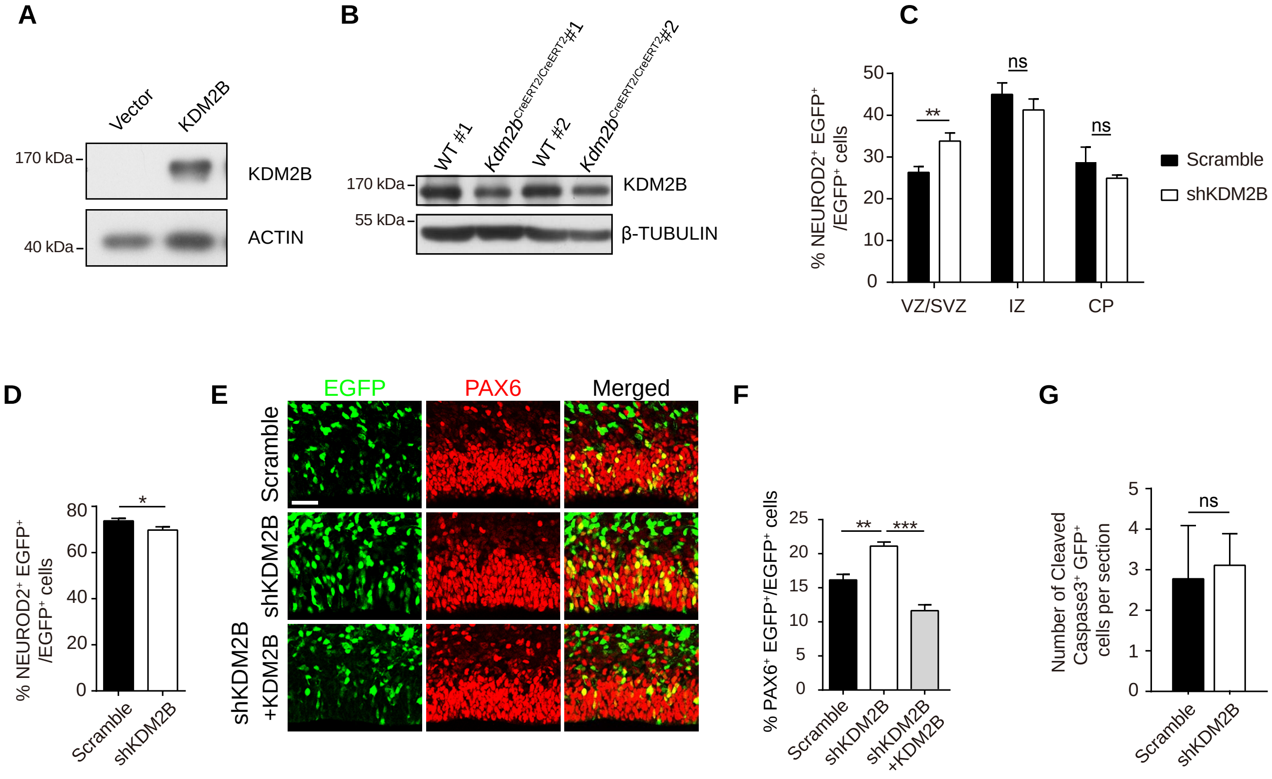
A
Vector
KDM2B
170 kDa
KDM2B
40 kDa
ACTIN
B
WT #1
Kdm2b CreERT2/CreERT2 #1
WT #2
Kdm2b CreERT2/CreERT2 #2
170 kDa
KDM2B
55 kDa
β-TUBULIN
C
% NEUROD2+ EGFP+ /EGFP+ cells
**
ns
ns
VZ/SVZ
IZ
CP
Scramble
shKDM2B
D
% NEUROD2+ EGFP+ /EGFP+ cells
*
Scramble
shKDM2B
E
EGFP
PAX6
Merged
Scramble
shKDM2B
shKDM2B +KDM2B
F
% PAX6+ EGFP+/EGFP+ cells
**
***
Scramble
shKDM2B
shKDM2B +KDM2B
G
Number of Cleaved Caspase3+ GFP+ cells per section
ns
Scramble
shKDM2B

Figure S6

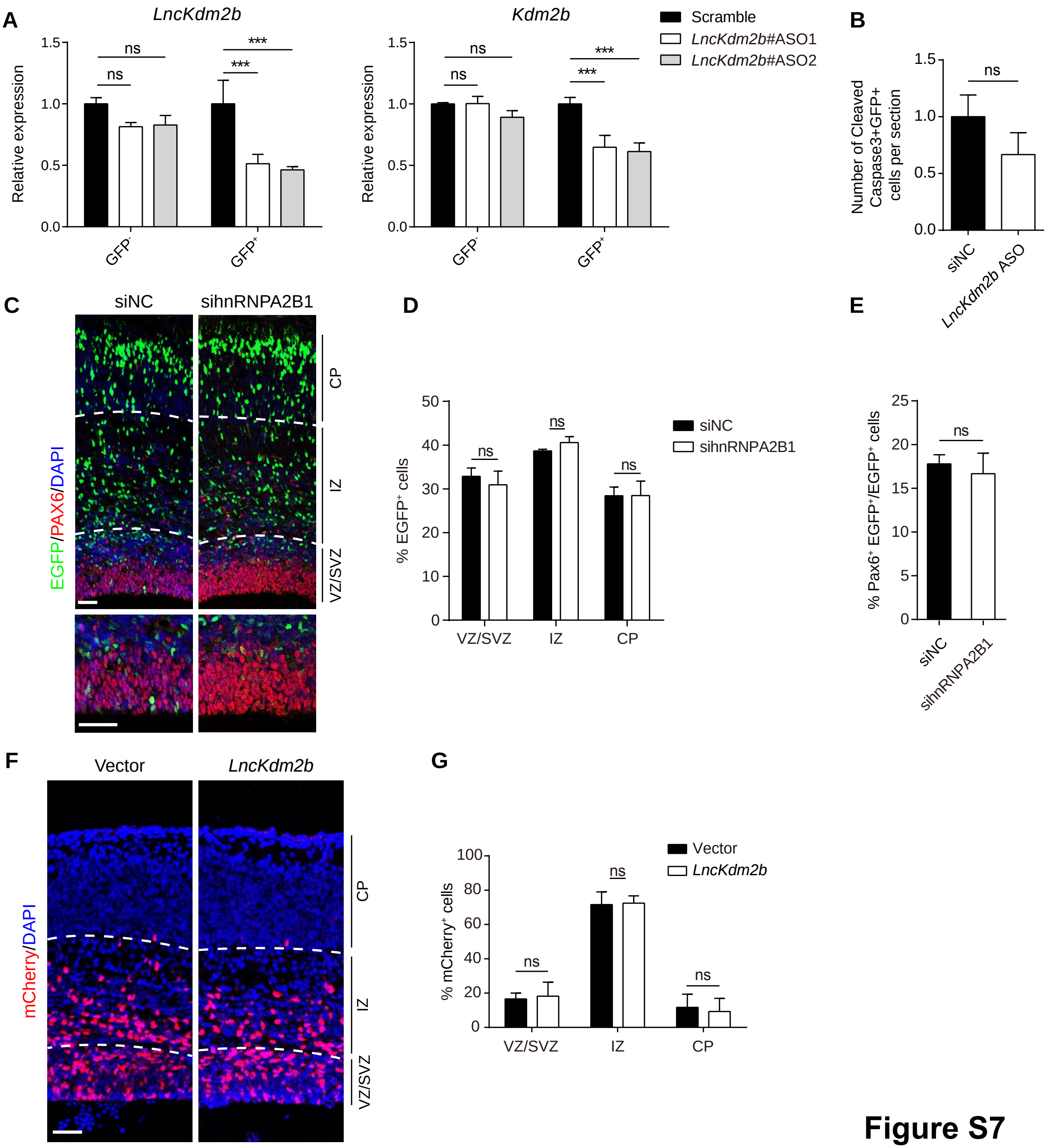
A
LncKdm2b
Kdm2b
Scramble
LncKdm2b#ASO1
LncKdm2b#ASO2
Relative expression
ns
***
GFP-
GFP+
B
Number of Cleaved Caspase3+GFP+ cells per section
siNC
LncKdm2b ASO
C
siNC
sihnRNPA2B1
EGFP/PAX6/DAPI
CP
IZ
VZ/SVZ
D
% EGFP+ cells
siNC
sihnRNPA2B1
E
% Pax6+ EGFP+/EGFP+ cells
F
Vector
LncKdm2b
mCherry/DAPI
G
% mCherry+ cells

Figure S7

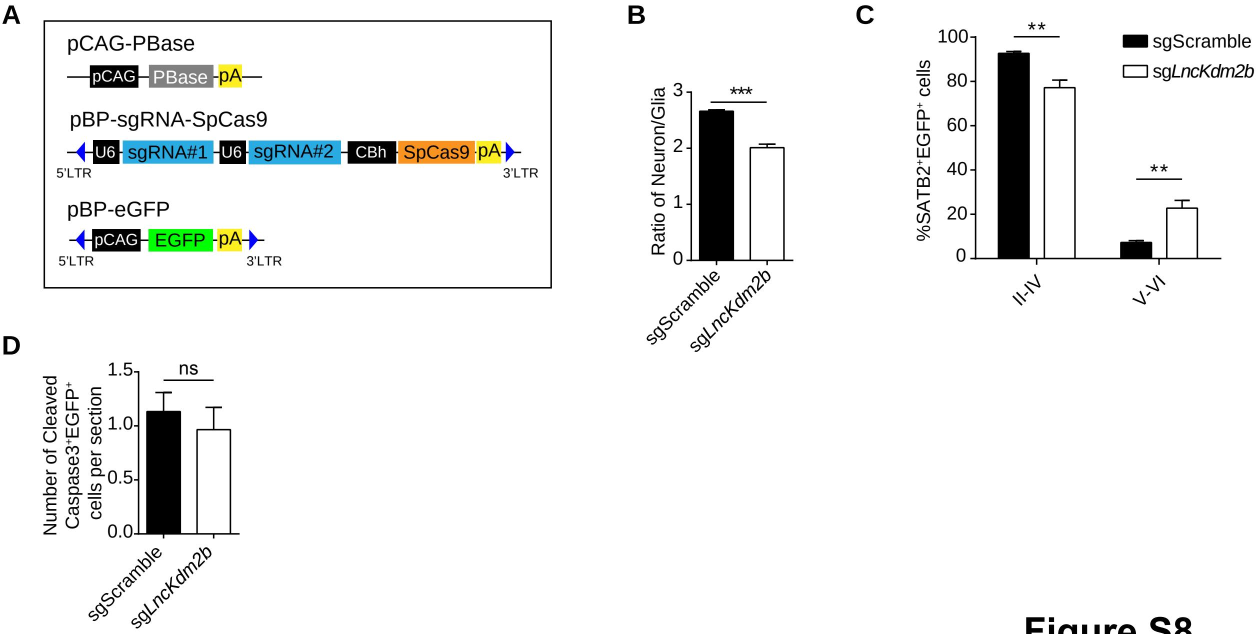
A
pCAG-PBase
pCAG
PBase
pA
pBP-sgRNA-SpCas9
U6
sgRNA#1
U6
sgRNA#2
CBh
SpCas9
pA
5'LTR
3'LTR
pBP-eGFP
pCAG
EGFP
pA
5'LTR
3'LTR
B
Ratio of Neuron/Glia
***
sgScramble
sgLncKdm2b
C
%SATB2+EGFP+ cells
**
sgScramble
sgLncKdm2b
II-IV
V-VI
D
Number of Cleaved Caspase3+EGFP+ cells per section
ns
sgScramble
sgLncKdm2b

Figure S8
